# Complex wall modeling for hemodynamic simulations of intracranial aneurysms based on histologic images

**DOI:** 10.1007/s11548-021-02334-z

**Published:** 2021-03-14

**Authors:** Annika Niemann, Samuel Voß, Riikka Tulamo, Simon Weigand, Bernhard Preim, Philipp Berg, Sylvia Saalfeld

**Affiliations:** 1 Faculty of Computer Science, Otto-von-Guericke University Magdeburg, Universitätsplatz 2, D-39106 Magdeburg, Germany; 2 Laboratory of Fluid Dynamics and Technical Flows, Otto-von-Guericke University Magdeburg, Magdeburg, Germany; 3 Forschungscampus STIMULATE, Magdeburg, Germany; 4 Department of Vascular Surgery, and Neurosurgery Research Group, Helsinki University Hospital and University of Helsinki, Helsinki, Finland; 5 Department of General, Visceral and Transplantation Surgery, Hospital of the University of Munich, Campus Grosshadern, Munich, Germany

**Keywords:** Intracranial aneurysms, Aneurysm wall, Histologic images, Structural simulation

## Abstract

**Purpose:**

For the evaluation and rupture risk assessment of intracranial aneurysms, clinical, morphological and hemodynamic parameters are analyzed. The reliability of intracranial hemodynamic simulations strongly depends on the underlying models. Due to the missing information about the intracranial vessel wall, the patient-specific wall thickness is often neglected as well as the specific physiological and pathological properties of the vessel wall.

**Methods:**

In this work, we present a model for structural simulations with patient-specific wall thickness including different tissue types based on postmortem histologic image data. Images of histologic 2D slices from intracranial aneurysms were manually segmented in nine tissue classes. After virtual inflation, they were combined into 3D models. This approach yields multiple 3D models of the inner and outer wall and different tissue parts as a prerequisite for subsequent simulations.

**Result:**

We presented a pipeline to generate 3D models of aneurysms with respect to the different tissue textures occurring in the wall. First experiments show that including the variance of the tissue in the structural simulation affect the simulation result. Especially at the interfaces between neighboring tissue classes, the larger influence of stiffer components on the stability equilibrium became obvious.

**Conclusion:**

The presented approach enables the creation of a geometric model with differentiated wall tissue. This information can be used for different applications, like hemodynamic simulations, to increase the modeling accuracy.

## Introduction

An important aspect in the research of intracranial aneurysm (IA) development and rupture risk assessment is the simulation of blood flow inside the aneurysm including the analysis of hemodynamic characteristics. In ruptured IAs, a larger mean velocity, mean and maximum (flow-induced) wall shear stress and mean and maximum oscillatory shear index were found [7]. Other studies show contradicting results: Jou et al. [14] reported similar maximal wall shear stresses in ruptured and unruptured aneurysms and found that a large part of ruptured aneurysms had low wall shear stress. Cebral et al. [3] associated low flow conditions with degenerative histologic changes of the aneurysm wall. Model simplifications impair the results of numerical simulations, e.g., related to cerebral flow rates or vital parameters under physical activity [2].

The majority of those studies based on hemodynamic simulations assume arterial vessel walls to be rigid, i.e., with infinite resistance. This is very common as it reduces the modeling effort and avoids the need for wall properties which can hardly be determined. Wall movement is considered to be small and, as a result, has a minor impact on hemodynamic parameters [6]. However, only the inclusion of the wall itself in the simulation model enables the analysis of the intramural wall stresses and thus the location where rupture takes place. Intracranial aneurysm walls are characterized by strong heterogeneity [10]. In a previous work, the inclusion of patient-specific vessel wall thickness for fluid simulation yields much higher wall stress values for the IA’s rupture site [26]. The wall thickness was extracted with an industrial micro-CT scanner. However, no patient-specific wall properties were assigned, and the whole wall was modeled as homogeneous structure. With the help of histology, a classification of the wall composition can be conducted. This includes the analysis of local properties like plaques or collagen deposits.

Even before considering patient-specific wall thicknesses in aneurysms, earlier studies focused on the wall thickness of vessels. Bazilevs et al. [1] generated a vessel model with variable wall thickness. Voß et al. [27] extracted a vessel bifurcation of the circle of Willis postmortem and scanned it using optical coherence tomography. The images resolved the local wall thickness and were combined to a 3D model. This model was compared to others with constant or diameter-dependent wall thickness using fluid–structure interaction. The results revealed a strong impact on the local wall stress distribution. In finite element models of the artery wall different plaque types (hypercellular, hypocellular and calcified) had a significant influence on the stresses inside the wall. The wall stress was reduced with the stiffer calcified plaque compared to cellular plaques [24]. Fortunato et al. [8] found that calcification influences the behavior of cerebral aneurysm tissue. They generated an aneurysm model with calcification from micro-CT and multiphoton images. Based on these studies, we expect the stress inside the aneurysm wall to be influenced by the different tissues occurring in the wall. In this study, we present detailed tissue segmentation and aneurysm wall model generation from histologic images. These are the prerequisites for future studies on 3D aneurysm wall analysis. We use postmortem collected whole aneurysms instead of intraoperative samples, which are restricted to the aneurysm dome. The data collection involved the analysis of 200 human cadavers by the Forensic Institute to find and extract intracranial aneurysms with their parent vessels. This allows analysis of the transition from neck to dome. The focus of this work is the tissue segmentation and model generation.

To understand the processes of wall degeneration, aneurysm development and attributes of ruptured wall-like calcification and fiber direction are combined with simulations. This requires the extraction of wall attributes and the combination with a 3D model.

The evaluation of histologic data is usually carried out in 2D. 3D models have to be created based upon the 2D data. Recently, Tomoaki et al. [23] have reconstructed a 3D model of an anterior anorectum from histologic images stained with Elastica van Gieson. After manual registration and manual segmentation, they used commercial 3D reconstruction software. Kugler et al. [17] described a landmark-based approach to register histologic images of different stains to generate a 3D histologic image. Based on multiple histologic datasets, Kraut et al. [16] constructed a 3D atlas of the human thalamus using affine registration and elastic deformation to refine a model from one dataset. In our previous work, the reconstruction of a 3D IA model from several 2D histologic images included a multilayer segmentation in intima, media and adventitia [21]. As these layers may dissolve during the progression of IAs, this segmentation may not be applicable for all IAs. Frösen et al. [9] and Kataoka et al. [15] showed that changes in the aneurysm wall (de-endothelialization, luminal thrombosis, smooth muscle cell proliferation, T-cell and macrophage infiltration) precede rupture of IAs. In addition, rupture was associated with structural damages in the aneurysm wall and inflammatory cell invasion. Costalat et al. [5] found that the wall of unruptured IAs is more rigid than the wall of ruptured ones. Furthermore, the local stiffness depends on the load direction [25].

Frösen et al. [9] introduced four intracranial aneurysm wall types: (A) linearly organized smooth muscle cells and intact endothelium, (B) thickened wall, with disorganized, proliferating smooth muscle cells, occasionally bearing a luminal fresh or organizing thrombus, (C) thick but decellularized of former myointimal hyperplasia (MH) or organized thrombus and (D) very thin wall, decellularized, with an organized luminal thrombus. This classification was used in a previous aneurysm model [21]. It has several limitations: The wall types are only defined for the aneurysms and not for the parent vessel and in aneurysms the wall types can overlap, resulting in an ambiguous segmentation. To use characteristic tissue values for structural simulations (like Young’s modulus), the segmentation should be unambiguous.

In some aneurysms, a thrombus can be found and thus should be included in the aneurysm model. Traditionally, a fresh thrombus can be divided into white thrombi (mainly composed of fibrin and platelet aggregates) and red thrombi (mainly being enriched in fibrin and erythrocytes) [18]. In this regard, Wilson et al. [28] analyzed thrombi in abdominal aortic aneurysms. They found that the fresh thrombus of aneurysms is biologically active and cannot be correctly modeled as homogeneous inert material. Older thrombus becomes organized. Intracranial aneurysms contain thrombus of different states [9, 10]. With further insight into the changes in the aneurysm wall tissue, treatment might be improved with pharmacological therapy [9].

In the present study, a new model based on different tissue textures is introduced. It is based on histologic image data and applied to structural simulations of two aneurysm segments. The tissue analysis is more detailed than the previous classification by Frösen et al. [9] based on combinations of these features and is suitable for the aneurysm as well as the parent vessel.

## Materials and methods

**Fig. 1:**
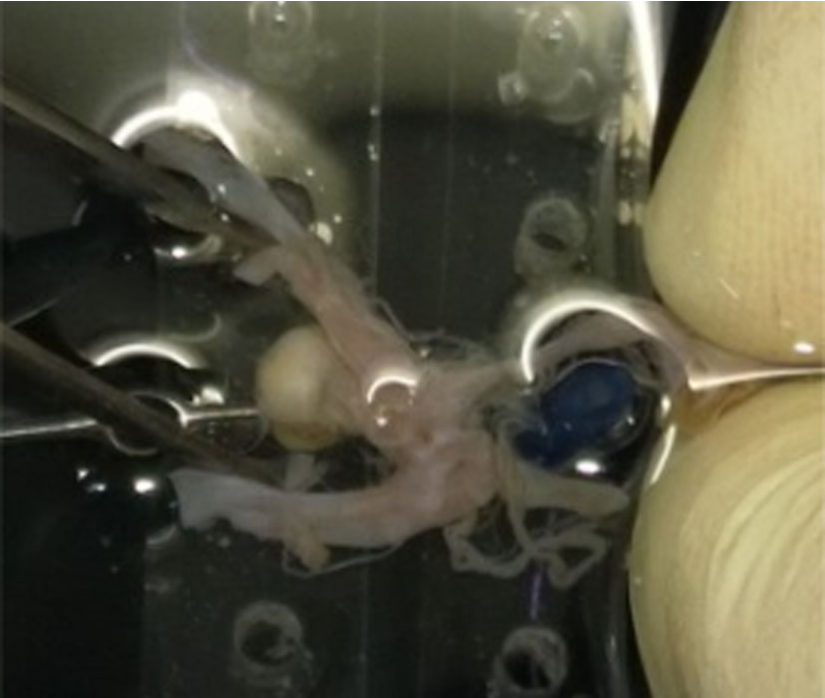
Postmortem collection of intracranial aneurysms

**Fig. 2:**
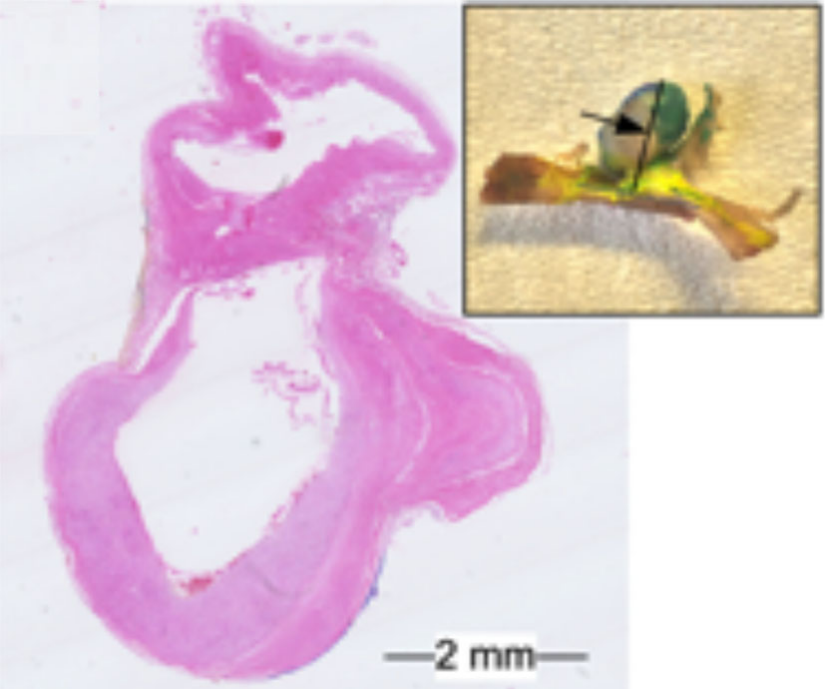
Extracted intracranial aneurysm and histologic image

For this study, three IA datasets were available and were used for tissue classification and subsequent segmentation of different tissue parts. The data were collected in a pathological study where 200 human cadaver were analyzed. If present, intracranial aneurysm were extracted as shown in Fig. 1 and Fig. 2. Segments of these datasets were then used to create reduced 3D models to prove the applicability for structural simulations.

The aneurysms were collected postmortem in cooperation with the Forensic Institute of the University Hospital Magdeburg with approval of the local ethics committee. After harvesting, the specimens were stained with hematoxylin and eosin (H&E). Each specimen contained the IA dome, neck and the parent vessel. The specimens were embedded in paraffin, sliced axial to the aneurysm sac and scanned with a Hamamatsu Nanozoomer (Hamamatsu Photonics, Hamamatsu, Japan) resulting in between 51 and 66 slices each. The $2-{\upmu }\hbox {m}$-thick slices are $100\,{\upmu }\hbox {m}$ apart. More information about the image acquisition can be found in [11].

**Fig. 3:**
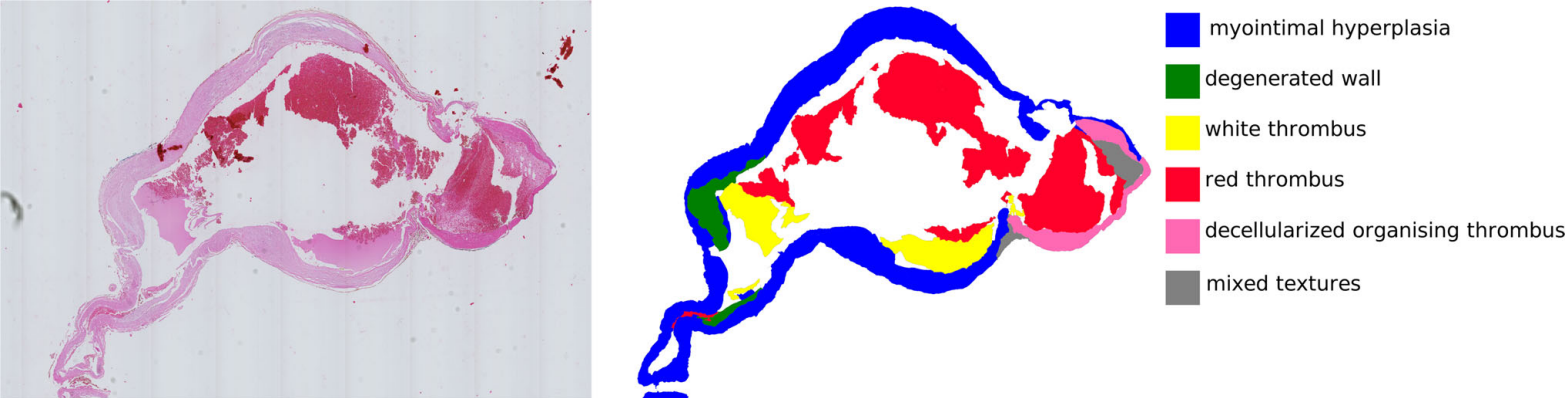
Depiction of the histologic 2D image of a H&E stained intracranial aneurysm section and corresponding segmentation. Six different tissues were identified in the image: blue: myointimal hyperplasia, green: degenerated wall, yellow: white thrombus, red: red thrombus, pink: decellularized organizing thrombus and gray: mixed textures

### Tissue classification in histologic images

The digitalized whole slide images were utilized for tissue texture classification; see Figs. 3 and  5. Taking into account the versatility of the local histologic changes in the aneurysm wall, a non-overlapping segmentation solely based on the images was chosen.

**Fig. 4:**
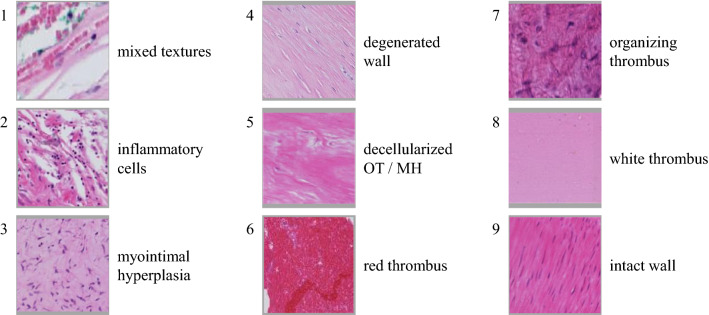
Detailed, non-overlapping tissue texture classification for histologic images of intracranial aneurysms

Based on the three IA datasets, we defined nine tissue texture classes visible in the H&E stained whole slide images. This categorization was discussed and refined with two medical experts (R.T. and S.W.) and is presented in Fig. 4. The classes can be described as follows:

- *Mixed textures (1) * include all areas that cannot be classified into one of the other classes. This includes unusual findings and areas where a reliable identification is not possible. In the patch in Fig. 4, infiltrating red blood cells between connective tissue bundles are shown.
- *Inflammatory cells (2)* show regions with an increased amount of inflammatory cells.
- *Myointimal hyperplasia (3)* (MH)
- *Degenerated wall (4) * shows wall tissues of the aneurysm wall with signs of wall degeneration, defined as loss of mural cells.
- *Decellularized organizing thrombus (OT)/MH (5)* shows decellularized tissue. In histologic images, the origin of this tissue is difficult to determine.
  Three textures are used to describe the thrombus:
  - *Red thrombus (6)* (RT), i.e., a fresh thrombus with a lot of red blood cells.
  - *Organizing thrombus (7)* (OT).
  - *White thrombus (8) *, i.e., a thrombus made of fibrin with very few red blood cells.
- *Intact wall (9)* shows intact wall tissue with linearly organized mural cells, of most likely smooth muscle cells based on the morphology and location.

**Fig. 5:**
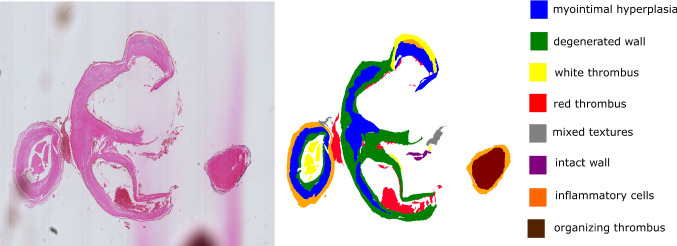
Depiction of another histologic 2D image of a H&E stained intracranial aneurysm section and corresponding segmentation. Eight different tissues were identified in the image: blue: myointimal hyperplasia, green: degenerated wall, yellow: white thrombus, red: red thrombus, gray: mixed textures, purple: intact wall, orange: inflammatory cells and brown: organizing thrombus

### 3D model generation

**Fig. 6:**
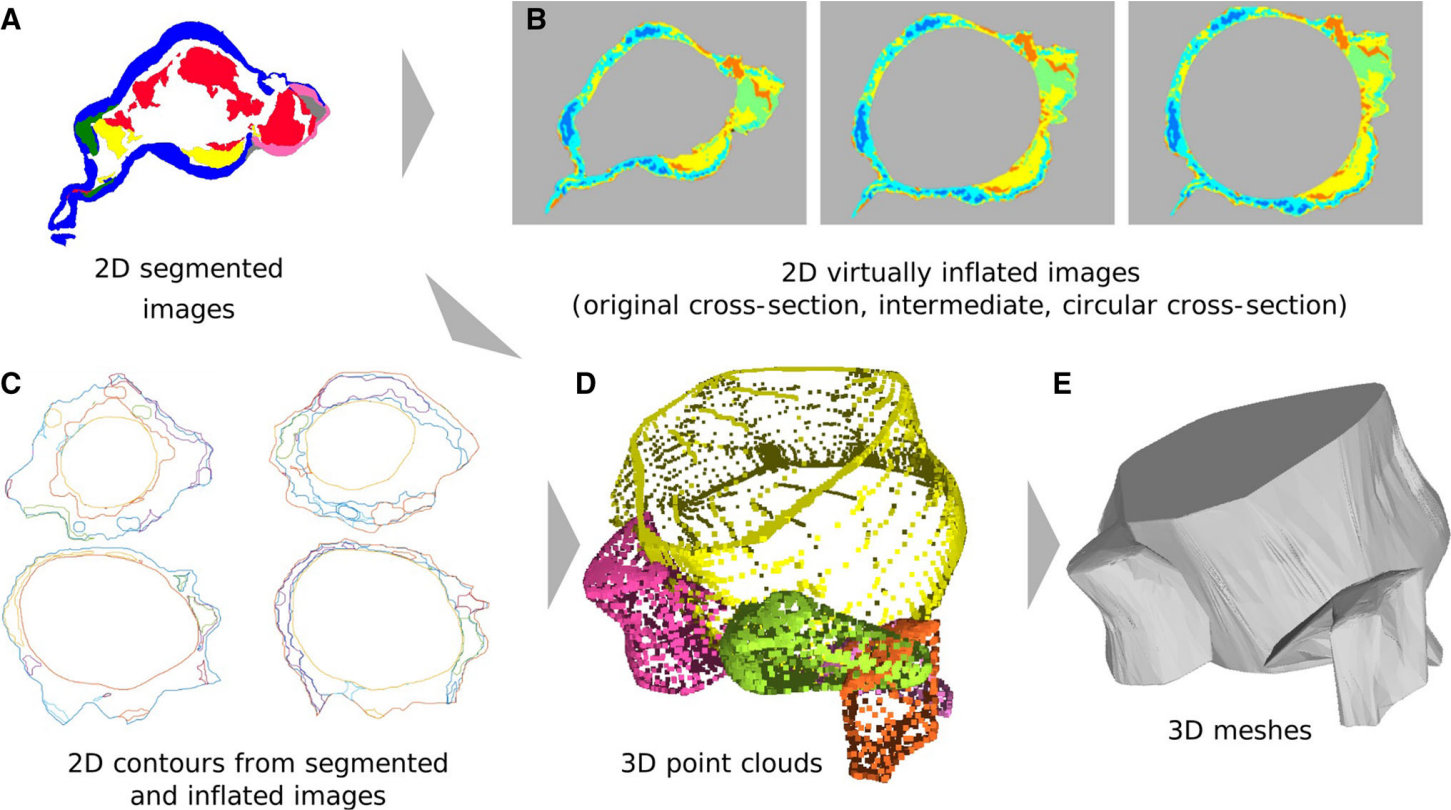
Concept for the reconstruction of 3D meshes for the patient-specific wall thickness and wall composition based on the segmented 2D histologic images. **a** image segmentation, **b** virtual inflation of segmented images, **c** contours derived from segmented and inflated images, **d** contours combined to 3D point clouds, **e** surface meshes from point clouds

Based on the histologic whole slide images and the presented tissue characterization, we carried out manual segmentations of the different tissue classes and built 3D models. Our pipeline is presented in Fig.  6.

In order to use a surface model from an arbitrary image modality for any kind of fluid or structural simulation, it must meet certain requirements. The surface must be closed and should not contain any non-manifolds or duplicate faces for easy processing. In addition, unrealistically sharp edges must be avoided; otherwise, this can lead to singular points with locally wrong solutions. But at the model edges, which represent a system boundary, a clear edge is necessary. These are, for example, the virtual slice planes where a model section is cut out from the surrounding structure. Therefore, a separate boundary condition is applied at these cut planes, which requires a clear demarcation from other boundaries like mechanically free surfaces.

The first step is the segmentation of the histologic images. Different segmentations were carried out manually: a segmentation of the inner and outer contour of the IA’s walls and a segmentation of the different tissue classes, as described in “Tissue classification in histologic images” section. While the histologic images contain small details, like cell nuclei, the segmented images comprise larger, uniformly colored areas. Therefore, the image resolution is reduced by 85%. On the segmented images, a virtual inflation as described by Glaßer et al. [11] is performed. For the virtual inflation, the inner contour is projected to a circle in order to account for deflation during the postmortem explanation of the specimens. We do not use the perfect circular inner contour, but use the interpolation step of 4/10, which was empirically determined to be a suitable interpolation step.

After the virtual inflation, the contours of the segmented areas are determined. For each tissue class, a binary mask is generated. Next, a connected component analysis is applied to the binary mask and the boundaries of each component are extracted with the Moore neighbor tracing algorithm using Jacob’s stopping criteria [12]. As a result, we obtain closed 2D contours for each 2D histologic whole slide image and the comprised tissue categories.

The next step is a point cloud generation from these 2D contours that represent different tissue types. During the slicing and scanning of the slides, the orientation of each individual histologic slice can vary. Therefore, an affine registration using the coherent point drift algorithm [19] with up to 40 iterations is carried out. After registration, the algorithm searches for corresponding contours in consecutive slices. The contours are re-sampled to have the same number of points as the contours of the previous slide. The distance between the centers of two contours as well as the average distance between corresponding points of two contours is calculated. Only contours of the same tissue class are considered for matching. Matching contours are summarized in 3D point clouds, where the z-coordinate depends on the slice number and slice distance.

**Fig. 7:**
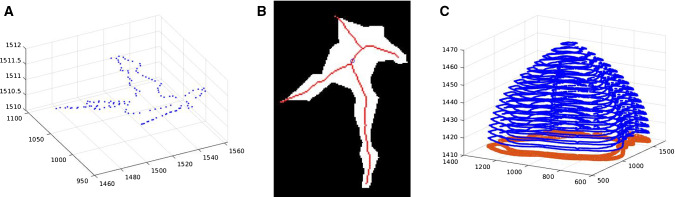
**a** Last contour of a tissue segment (contour of the tissue in the last slide where this tissue segment was visible) as 3D point cloud; **b** corresponding binary image, red line: points left after thinning, blue circle: center of the contour; **c** example of a dome added to a contour in 3D, red: top contour blue: added points for the dome

The resulting point clouds have flat endings. While it is not visible in the data, we assume that abrupt changes are uncommon in human tissue. To generate a realistic model and avoid artifacts during simulation due to unnatural sharp edges, a cap is added to the start and end of the point clouds. The first and last contour of a point cloud are determined by the smallest and largest z-values, respectively. In addition, their midpoints are determined. While keeping the midpoint of the contour constant, several smaller contours are generated and added above/below the last/first contour. The factor for decreasing the contour is based on a parabolic function, and therefore, the resulting smaller contours form a cap. For this procedure, the midpoint has to be inside the contour. To ensure this, the contour is transformed to a binary image and a thinning algorithm yielding the centerline of the shape is applied [12]. The points of this centerline are the candidates for the midpoint of the contour. The point with the largest distance to the nearest contour point is chosen as the midpoint (see Fig. 7).

In the last step, each point cloud is converted to a mesh by iterative fitting a start mesh to the points [20]. Due to tears in the tissue, folding of the tissues and impurities the contours from the images and the meshes might contain some noise. The meshes were manually smoothed to correct these problems. This results in meshes of the inner and outer contour and several meshes of larger regions of the same tissue type. Small intersections of the meshes may occur due to the mesh generation and smoothing. This can cause problems in applications like structural simulations. Boolean operations were used in this study to clear intersections and create distinct interfaces between meshes.

A structural simulation is generally based on simplifications and modeling assumptions. The solution is calculated only at discrete points. The number of these solution points depends on the spatial discretization and affect the computational effort. Therefore, the underlying surface model should only contain that level of detail, which can be spatial resolved by the simulation. In this investigation, structures smaller than $1\,\hbox {mm}^{3}$ are neglected.

### Structural simulation

The high level of detail provided by the tissue modeling and resulting large number of meshes are challenging for simulations. In order to demonstrate a possible application of such detailed models, we evaluated its impact based on structural simulations. Two reduced models consisting of three (Model A) and ten slices (Model B) are imported in the commercial software STAR-CCM+ 13.06 (Siemens Product Lifecycle Management Software Inc., Plano, TX, USA). In both cases, one simulation includes several meshes and different tissue classes representing the heterogeneous wall state. Table 1 lists the tissue classes (only those included in the reduced models, some tissue classes did not occur in the slices used for the reduced models) and their elastic properties. A linear elastic material is considered (Poisson’s ratio of 0.45 for all classes). Each configuration is compared to a homogeneous one which consists of only one tissue class, intact wall.

**Table 1:** Young’s moduli of all tissue classes used in the simulation. Values for classes without literature information were approximated based on their visual texture by a domain expert. (tissue classes 2, 4 and 7 were not present in the model)

Tissue class	Young’s modulus (kPa)	Reference
1 - Mixed textures	85.17	(Average of tissue 8 and tissue 9)
3 - Myointimal hyperplasia	109.54	(1/4 of tissue 8, 3/4 of tissue 9)
5 - Decellularized OT/MH	72.99	(Average of tissue 3 and tissue 8)
6 - Red thrombus	18.22	(Half of tissue 8)
8 - White thrombus	36.44	Noble et al. [22]
9 - Intact wall	133.9	Noble et al. [22]

**Fig. 8:**
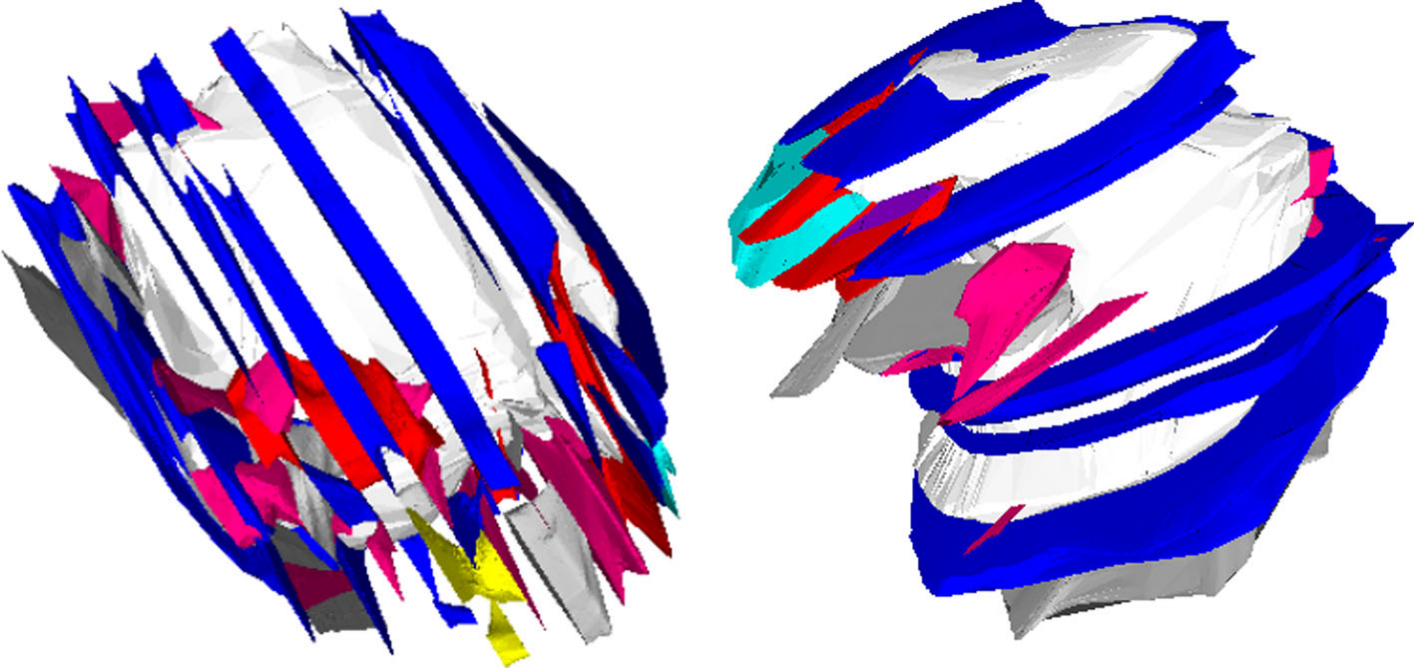
Resulting model for one of the aneurysm datasets (without outer mesh) from two different views. White: mesh of inner wall, colored meshes: selected different tissue segments; the model is based on 22 consecutive slices, distance between slices $100\,\upmu \hbox {m}$, image resolution per slice around $18500 \times 6000$

The reduced models are discretized by tetrahedral finite elements cells with linear basis functions. Large geometric deformations are enabled. The following boundary conditions are defined: The outer wall is a free surface, the inner wall is subjected to the intravascular pressure, and the slice area has a symmetry condition to mimic the link to the connected tissue.

## Results

Based on the proposed pipeline, highly resolved histologic images of IA wall can be converted to a 3D model preserving the wall composition. Utilizing the introduced classification of tissues textures into nine different types a high level of heterogeneity is maintained. Each IA model consists of two global meshes that mark the inner and outer wall. In between them, a number of smaller adjacent meshes representing the individual sections of different tissue types are located. Finally, the models meet all requirements to be applied to various visualization, quantification or simulation purposes.

Most tissue segments can only be traced a few slices at most, resulting in small meshes. While some tissue changes are found in the parent vessel, most changes occur in the aneurysm itself. The model for the aneurysm consists of 94 meshes of tissue segments, a mesh of the inner aneurysm wall and a mesh of the outer aneurysm wall. A selection of these meshes is shown in Fig. 8. The whole model was generated based on 22 consecutive slices. As the mesh generation requires presence of the tissue in consecutive slices, smaller tissue segments are not captured and some volumes between the outer and inner mesh are not covered by tissue meshes.

**Fig. 9:**
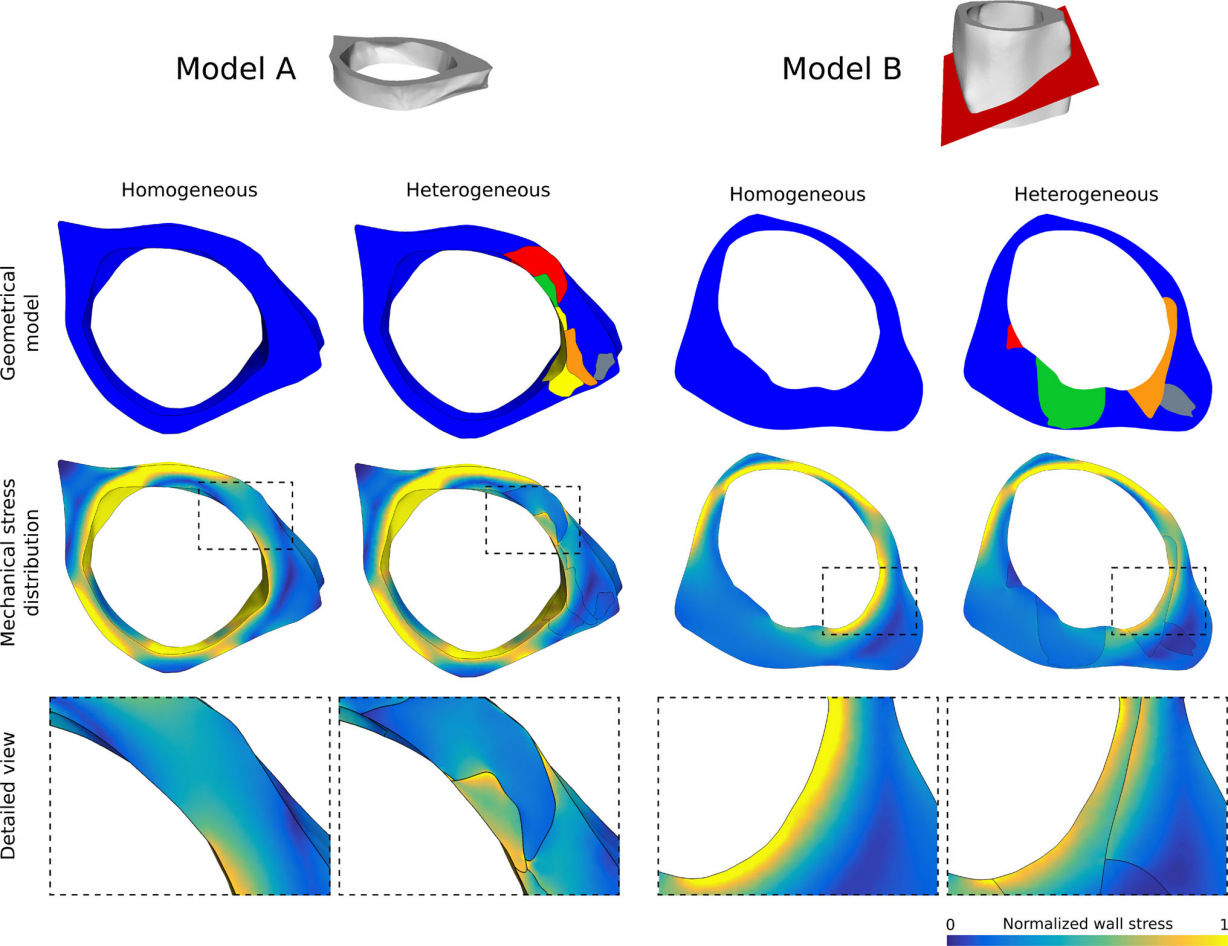
Reduced models A and B of the patient-specific vasculature as homogeneous configuration and heterogeneous configuration. Top: model composition from different tissue classes; middle: resulting mechanical wall stress distribution of the structural simulations of the wall under intraluminal blood pressure; bottom: detailed view of the local wall stress distribution

**Fig. 10:**
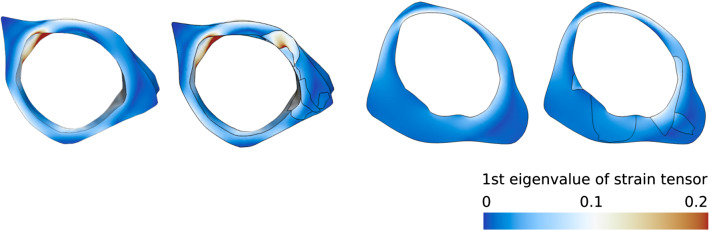
Strain fields of the models

Figures 9 and  10 show the results of the structural simulation based on the reduced models A and B. On the left, the homogeneous configuration is shown. On the right, the heterogeneous configuration consisting of up to seven different tissue classes is depicted. Wall stress patterns are identical in regions that consist of intact tissue only. The clustering of several tissue classes causes a more heterogeneous wall stress distribution. Due to varying mechanical tissue elasticity, the individual components are able to compensate for the load at different levels. The stiffer components have a larger influence on the stability equilibrium than the flexible ones. This is particularly obvious in the stress transitions at interfaces between neighboring tissue classes. The intraluminal pressure leads to local deformations of the aneurysm wall and mechanical stresses inside the tissue. Increased pressure results in increased wall stress up to the maximum wall strength. Stress above this limit will cause rupture.

## Discussion

We defined nine classes of tissue textures found in histologic images of intracranial aneurysms, which can be identified solely based on the pattern. Based on the data collected from serial sections we were able to create a 3D model representing these different tissue classes in intracranial aneurysm and show that variance of tissue in the structural simulation affect the simulation result.

This work only includes H&E stained images. A variation of stainings would be desired to get more reliable information about the different tissues and cell types. However, the specimens were acquired postmortem and are only very rarely available. The segmentation could be further refined. For example, without cell-type-specific stainings the definitive identification of inflammatory cells is complicated. Also, the *degenerated wall* class could be divided into adventitial and luminal side. Further analysis of the location of the defined tissue class within the aneurysm wall could allow division in *decellularized OT* and *decellularized MH*.

While histologic images provide an excellent in-plane resolution, the large gaps ($100\,\upmu \hbox {m}$ in this study) between the slices limit the resolution in the third direction. Furthermore, if the volume of a tissue part is too small compared to the gap between the slices, it may be only visible in one slice and cannot be traced in the previous or following slice. Nevertheless, textures could be mostly traced through at least a few slices.

The reconstruction of different tissues in IAs is complex due to the local remodeling and thrombosis of the wall, which change tissue locally over time and lead to a heterogeneous tissue class representation. Frequent changes in the tissue occur and large regions of the same texture are rare.

Thus, the expressiveness of the model is limited. In reality, the changes between tissues in the wall are more blurred than suggested by the clear borders presented between classes of the model. For the generation of meshes, it is necessary to define clear borders between tissue classes even if there is a smooth transition between them. In the future, this might be addressed by adding transition classes and creating volume meshes with cell-specific properties.

The tissue within a single tissue mesh is not further refined and modeled as uniform. More detailed information like fiber direction could further improve the model.

Here, the luminal shape has been artificially inflated round. In reality the aneurysm lumen comes with different irregularities. In vivo information of the lumen shape could improve the aneurysm model.

Aneurysm walls are pathological and therefore different from healthy vessel walls. Accordingly, the models for healthy tissues are only partially transferable to intracranial aneurysms. Therefore, detailed and individual modeling is essential to assess the individual strength of an aneurysm. In the future, this could comprise the differentiation into aneurysm-specific tissue types, the morphological condition and mechanical properties, including failure limits. The simulation results of the reduced models serve as examples, how these aspects affect the simulation outcome. The more pronounced the heterogeneity of the wall structure, the more heterogeneous is the distribution of wall stresses. Realistic and reliable simulations require advanced wall models. They are based on the simple concept that aneurysm rupture represents the moment of wall stress exceeding wall strength. Therefore, knowing the wall morphology, local mechanical properties and loads (intravascular pressure and flow-induced shear) can lead to detailed models of rupture probability. This study represents early steps toward such modeling approaches. The presented finite element simulations have limitations. Fiber orientation or complex material behavior are not represented by the isotropic, linear elastic model. In addition, the boundary/loading conditions are simplified. However, they do illustrate the difference between a homogeneous and a heterogeneous wall model. While this study focuses on the aneurysm wall only, future investigations might also compare aneurysm tissue and parent vessel tissue as well as resulting stresses.

Further validation of the model shape and wall thickness needs a reference model of the aneurysm before slicing. Unfortunately, that is not available for this dataset.

The detailed models presented here are based on histologic images. Currently used diagnostic images do not provide the same level of detail. The wall tissue thickness and calcification could be also captured with micro-CT [4]. This avoids the problem of deformation of the tissue during the slide preparation and image registration as necessary for the model based on histologic images. While the model generation is simpler, the information about the vessel wall tissue is very limited. However, new imaging techniques like optical coherence tomography might allow detailed vessel wall imaging in the future and make simulations with patient-specific wall composition possible [13].

The presented model generation could be useful for other aneurysms (e.g., aortic and popliteal aneurysm) as well as stenoses and plaques in coronary arteries.

## Conclusion

We presented a classification of intracranial aneurysm wall tissue textures in histologic images. Based on this classification, a pipeline for the generation of a detailed aneurysm model is described. The model consists of several meshes representing different tissue texture classes. These are used in structural proof-of-concept simulations. Stiffer components contribute more to the stability than flexible tissue, which leads to relevant differences between simulations of homogeneous and heterogeneous walls.
